# Targeting an Essential GTPase Obg for the Development of Broad-Spectrum Antibiotics

**DOI:** 10.1371/journal.pone.0148222

**Published:** 2016-02-05

**Authors:** Josephine A. Bonventre, Ryszard A. Zielke, Konstantin V. Korotkov, Aleksandra E. Sikora

**Affiliations:** 1 Department of Pharmaceutical Sciences, College of Pharmacy, Oregon State University, Corvallis, OR, 97330, United States of America; 2 Department of Molecular and Cellular Biochemistry, and Center for Structural Biology, University of Kentucky, Lexington, KY, 40536, United States of America; University of Lethbridge, CANADA

## Abstract

A promising new drug target for the development of novel broad-spectrum antibiotics is the highly conserved small GTPase Obg (YhbZ, CgtA), a protein essential for the survival of all bacteria including *Neisseria gonorrhoeae* (GC). GC is the agent of gonorrhea, a prevalent sexually transmitted disease resulting in serious consequences on reproductive and neonatal health. A preventive anti-gonorrhea vaccine does not exist, and options for effective antibiotic treatments are increasingly limited. To address the dire need for alternative antimicrobial strategies, we have designed and optimized a 384-well GTPase assay to identify inhibitors of Obg using as a model Obg protein from GC, Obg_GC_. The assay was validated with a pilot screen of 40,000 compounds and achieved an average Z’ value of 0.58 ± 0.02, which suggests a robust assay amenable to high-throughput screening. We developed secondary assessments for identified lead compounds that utilize the interaction between Obg_GC_ and fluorescent guanine nucleotide analogs, mant-GTP and mant-GDP, and an Obg_GC_ variant with multiple alterations in the G-domains that prevent nucleotide binding. To evaluate the broad-spectrum potential of Obg_GC_ inhibitors, Obg proteins of *Klebsiella pneumoniae* and methicillin-resistant *Staphylococcus aureus* were assessed using the colorimetric and fluorescence-based activity assays. These approaches can be useful in identifying broad-spectrum Obg inhibitors and advancing the therapeutic battle against multidrug resistant bacteria.

## Introduction

Antibiotic resistance is a natural phenomenon made more precipitous by the misuse of antimicrobial drugs, which has accelerated the appearance of drug-resistant bacteria (reviewed in: [1]). It is estimated that over two million people are diagnosed with antibiotic resistant illnesses in the United States alone each year, and that greater than 300 million cumulative premature deaths will result worldwide by 2050 [2]. Antibiotic resistant infections, such as those caused by *Clostridium difficile*, carbapenem-resistant *Enterobacteriaceae*, particularly *Klebsiella* species, and methicillin-resistant *Staphylococcus aureus* (MRSA), result in prolonged illness, with potentially greater mortality and morbidity, and added healthcare expenditure [3, 4]. Contributing to the complications of increased multi-drug resistance is the relative lack of novel antibiotics introduced to market [1, 4, 5]. The drought of new discoveries in the field is due in large part to the absence of many Big Pharma research and development programs in this area [6, 7]. Drug discovery remains an expensive and time-consuming endeavor, costing pharmaceutical companies millions of dollars to bring a compound from bench to market, and often these chemicals fail before entering clinical trials [7–9]. In addition, an effective antibiotic ideally cures an infection in a few doses, which makes the financial investment into the drug discovery process not as profitable as medication for chronic illnesses. Nevertheless, there is a pressing need to address the global antibiotic resistance health crisis, and the task of developing screening programs for novel drug targets now primarily rests on the shoulders of academia and small pharmaceutical companies [2, 4].

Investigating target molecules for the development of broad-spectrum antibiotics against drug-resistant bacterial infections is often more cost effective than developing a compound with activity against a single pathogen because of the wider clinical applications. As a result, biological targets that are essential to the physiology of the microorganism and conserved across various species are traditionally preferential drug candidates [6]. For example, inhibiting DNA replication, the ribosome function, or cell-wall biosynthesis has proven to be viable strategies [9, 10].

We have recently proposed the Obg protein (YhbZ, CgtA) as a molecular target for development of new therapeutic interventions against drug-resistant bacteria [11]. Obg and Obg-like proteins are highly conserved GTPases, present in bacteria, archaea, and eukaryota [12–15]. The bacterial Obg proteins have a unique structure (S1 Fig) containing highly conserved glycine-rich N-terminal domain, conserved GTP-binding domain, and a variable C-terminal domain [11, 12, 16, 17]. Few Obg proteins have been crystalized to date [16, 18], however, the N-terminus has thus far demonstrated the most favorable potential for protein-protein interactions [19–21].

G proteins typically display high affinities for nucleotides, low dissociation rates in the absence of exogenous exchange factors, and low intrinsic hydrolysis activity. In many eukaryotic Ras-like GTPases these properties are regulated by guanine exchange factors (GEFs), GTPase activating proteins (GAPs), and guanidine dissociation inhibitors (GDIs) [22]. Detailed biochemical analysis demonstrated, however, that Obg GTPases are clearly distinct. They display slow rate of GTP hydrolysis, micromolar binding constants for GTP and GDP, and rapid dissociation constants for either GTP or GDP, which are 10^3^–10^5^ faster than that of Ras-like GTPases. These biochemical features of Obg, if considered in the absence of potential GEFs, GAPs, and GDIs, suggest that Obg proteins act as intracellular sensors and their nucleotide-bound state is controlled by relative GTP/GDP concentration [12].

Obg homologs are essential for the survival of both Gram-positive and Gram-negative bacteria, including *Bacillus subtilis*, *Streptomyces coelicolor*, *Staphylococcus pneumoniae*, *S*. *aureus*, *Haemophilus influenzae*, *Caulobacter crescentus*, *Escherichia coli*, *Vibrio harveyi*, *V*. *cholerae*, and *Neisseria gonorrhoeae*, with depletion of the protein resulting in species-specific pleiotrophy [11, 23–29]. Bacterial Obg proteins have been associated with a variety of cellular functions, including ribosome biogenesis and maturation, DNA synthesis and replication, chromosomal segregation, regulation of the cell stress response, and recently, persistence in response to nutrient starvation [12, 13, 30]. Obg proteins bind not only GTP and GDP, but also the alarmone nucleotide, (p)ppGpp, which implies a role for this protein in the stringent response [11, 28, 31, 32]. Specifically, Obg-mediated persistence in *E*. *coli and Pseudomonas aeruginosa* requires (p)ppGpp, suggesting Obg may have a critical function in the failure of some antibiotic treatments [30].

Both the association between Obg and ribosome assembly and its proposed role in inducing bacterial multidrug tolerance make Obg a promising drug target [10, 11, 33]. To that end, our laboratory has targeted *N*. *gonorrhoeae* (gonococcus, GC) Obg, Obg_GC_, as a model protein for screening and identifying potential broad-spectrum inhibitors of Obg GTPase activity. GC, a Gram-negative bacterium, human-specific pathogen, and the etiological agent of gonorrhea, is listed among top three urgent antibiotic resistant threats according to the Centers for Disease Control and Prevention [34]. The sexually transmitted infection (STI) is a global health burden with 78 million of the estimated 498 million new cases of curable STIs that occur worldwide every year [35]. The disease causes cervicitis, urethritis, proctitis, conjunctivitis, or pharyngitis, and left untreated, leads to long-term health consequences including endometriosis, pelvic inflammatory disease, and infertility [36, 37]. At the turn of the 20^th^ century, GC infections were treated with penicillin, but over time, GC has acquired resistance to nearly all antimicrobials used in the clinic (reviewed in: [38, 39]). Currently, a vaccine for gonorrhea does not exist, and options for effective antibiotics are increasingly limited. Third-generation cephalosporin antibiotic regimens remain the final line of defense as multi-drug resistant GC isolates continue to be identified across the globe, threatening future treatment options [36, 38–41].

To address the dire need for alternative antimicrobial strategies against drug-resistant infections including gonorrhea, we developed an end-point Obg_GC_-GTP hydrolysis assay using the malachite green-based detection of the free phosphate as a read out. The assay was optimized and conducted in 384-well plates with a pilot screen of 40,000 small molecules. The Z’ values calculated from our initial screen of small molecules averaged 0.58 ± 0.02 over the course of five weeks, suggesting a robust, sensitive assay amenable to high-throughput screening (HTS). In addition, to eliminate nonspecific inhibitors and elucidate potential mechanism(s) of inhibition for lead compounds, we propose secondary Obg activity assessment methods based on the binding of fluorescent N-methyl-3’-*O*-anthranoyl-(mant)-guanine nucleotide analogs, mant-GTP and mant-GDP. A variant of Obg_GC_ with multiple alterations in the G-domains that is unable to bind both mant-GTP and mant-GDP was designed to serve as a positive control in the secondary assays. Finally, to test the broad-spectrum potential of Obg_GC_ inhibitors, the recombinant versions of Obg proteins from *Klebsiella pneumoniae* (Obg_KP_) and MRSA (Obg_MRSA_) were cloned, purified, and assessed using colorimetric and fluorescence-based activity experiments.

In summary, we report a strategy for targeting a pivotal GTPase Obg that can be used to identify and optimize broad-spectrum chemical inhibitors for the treatment of gonorrhea and other diseases caused by multidrug resistant bacteria.

## Materials and Methods

### Bacterial strains, plasmids, and growth conditions

The *E*. *coli* strains NEB5α and BL21(DE3) were used for genetic manipulations and protein overproduction, respectively. Bacteria were streaked from glycerol stocks stored at -80°C on Luria-Bertani (LB) agar supplemented with kanamycin (50 μg/mL) when needed. *E*. *coli* strains were cultured in LB medium at 37°C. Culture media utilized in this study were purchased from Difco.

### Construction of recombinant wild type and mutated version of Obg_GC_

Genomic DNA was isolated from GC FA1090, a clinical isolate (1–007) of *K*. *pneumoniae* and MRSA (ATCC 25923) using Wizard Genomic DNA Purification Kit (Promega, Madison, WI). Construction of recombinant wild type Obg_GC_ containing C-terminally attached 6×His-tag, His-Obg_GC_, was described previously [11]. To generate *Obg_GC_, a DNA fragment containing *obg* gene from GC FA1090 (NGO1990) with designed mutated sites within G domains T192S, T193S, A212L, D213E, P215H, N284Q, K285R, S329V and 6×His-tag was synthesized as gBlock Gene Fragment by Integrated DNA Technologies (IDT DNA, Coralville, IA). The gBlock fragment was cut with XbaI and HindIII and cloned into similarly digested pET-Obg_GC_ [11] to yield pET-*Obg_GC_. The presence of the desired mutations was confirmed by DNA sequencing at Center for Genome Research and Biocomputing at Oregon State University.

To create C-terminal 6×His fusion of *obg* from MRSA, the *obg* gene was amplified using primers obg (MRSA)-f (GATCCCATGGTTGTCGATCAAGTCAAAATATC) and obg (MRSA)-r (GACTAAGCTTCGCTCCTATTCAACGAATT). The resulting PCR product was digested with NcoI and HindIII and cloned into similarly treated pET-28a to create pET-Obg_MRSA_.

To create plasmid pKV1414 bearing Obg_KP_, the DNA fragment corresponding to residues 1–392 was PCR-amplified from *K*. *pneumoniae* genomic DNA using primers Obg_kpn_F1Nde (GAGACATATGAAGTTTGTAGATGAAGCAAC) and Obg_kpn_R392Hind (CTCAAGCTTAGCGTTTGTAGATGAATTCG), digested with NdeI and HindIII, and cloned into a modified pCDF-Duet1 vector (Novagen/Merck KGaA, Darmstadt, Germany), to append an N-terminal 6×His tag and a tobacco etch virus (TEV) protease cleavage sequence.

### Purification of *Obg_GC_, Obg_KP_ and Obg_MRSA_

All recombinant proteins were expressed using the *E*. *coli* BL21(DE3) as a host and purified as follows. Bacteria harboring pET-OBG_GC_, pET-*Obg_GC_, pKV1414, or pET-Obg_MRSA_ were cultured in LB supplemented with kanamycin at 37°C until the cultures reached OD_600_ of ~ 0.5, and the gene expression was induced by the addition of isopropyl-β-D-thiogalactoside (IPTG) to a final concentration of 1 mM. Cells were pelleted 3 h after induction and resuspended in lysis buffer (20 mM Tris-HCl pH 8.0, 10 mM imidazole, 500 mM NaCl). The cells were lysed by passing the suspension four times through a French pressure cell press at 12,000 psi. Bacterial debris was removed by centrifugation and filtered through 0.45 μm filter (VWR, Radnor, PA). Cell lysates were applied on 5 mL Bio-Scale™ Mini Profinity™ IMAC Cartridges (Bio-Rad, Hercules, CA) connected to NGC Chromatography System (Bio-Rad, Hercules, CA) equilibrated with 5 column volumes of lysis buffer. Column was washed with 8 column volumes of lysis buffer and bound protein was eluted with buffer containing 20 mM Tris-HCl pH 8.0, 250 mM imidazole, and 500 mM NaCl. Fractions containing Obg were pooled and concentrated using Vivaspin 20 (MWCO 10,000) spin concentrators (GE Healthcare Bio-Sciences, Pittsburgh, PA). Concentrated fractions were applied on HiLoad 16/600 Superdex 75 column (GE Healthcare Bio-Sciences, Pittsburgh, PA) and resolved using buffer containing 20 mM Tris pH 8 and 100 mM NaCl. Subsequently, samples containing the respective Obg variants were pooled and concentrated using Vivaspin 6 (MWCO 10,000) spin concentrators (GE Healthcare Bio-Sciences, Pittsburgh, PA).

Protein concentrations were determined using the Bradford method with a Protein Assay Kit (Bio-Rad, Hercules, CA). Glycerol was added to purified proteins to a final concentration of 10% and proteins were stored at -80°C.

### Assay optimization

A colorimetric GTPase assay was developed to screen for Obg inhibitors. The assay was based on the molybdate/malachite green detection method [42] and measures free phosphate in the solution following the addition of the BIOMOL® Green reagent (Enzo Life Sciences, Farmingdale, NY). The reaction was optimized for Obg_GC_ and GTP concentration in buffer A containing 50 mM Tris pH 8.0, 2 mM dithiothreitol, 1 mM ethylenediaminetetraacetic acid (EDTA), 50 mM KCl, 10% (wt/vol) glycerol and 10 mM MgCl_2_.

First, the optimal protein concentration for the assay was evaluated using 1, 2, 5, or 10 μM Obg_GC_ and 125 μM GTP with incubation time of 6 h at 37°C. Next, 5 μM Obg_GC_ was incubated with 125, 250, 500, 750 or 1000 μM GTP. At the end of each incubation period, BIOMOL Green was added at twice the volume of the reaction to quench it, and samples were allowed to incubate for 30 min at room temperature. The colorimetric measurements were performed at 620 nm using the BioTek Synergy HT Multi-Detection Microplate Reader (BioTek, Shoreline, WA). Absorbance values were related to the amount of phosphate released during Obg-mediated GTP hydrolysis. Ultimately, 5 μM Obg_GC_ and 250 μM GTP were selected, and a time-course was performed to measure phosphate release over time. The Km for Obg_GC_ was determined using 10, 25, 50, 100, 250, 500, 1000 μM GTP during a 6 h period.

To determine the optimal incubation length and temperature to be used for the primary screen of compound libraries, nine identically loaded 384-well plates with 25 μl reactions were incubated under three conditions: 6 h at 37°C, 18 h at 37°C, and 18 h at room temperature (3 plates each). In addition to the wells with complete reaction mixture (RM) containing 5 μM Obg_GC_, 250 μM GTP in buffer A (columns 2–22), each plate accommodated a background control comprising of RM lacking Obg_GC_, (column 1) and a positive control (RM without MgCl_2_; columns 23–24). The amount of free phosphate present under each condition was measured using BIOMOL Green as described above, and the difference between high-activity wells and low-activity wells was evaluated. Incubation for 18 h at 37°C was ultimately selected for future studies.

Obg_GC_ activity was tested for solvent tolerance and sensitivity to chelators. Obg_GC_ was incubated with various concentrations of dimethyl sulfoxide (DMSO), 0, 0.5, 1, 2, 3, 4, and 5%, in RM on 384 well plates as outlined above to determine if DMSO interferes with Obg-mediated GTP hydrolysis. The assay was incubated and the amount of free phosphate was measured as described above. The effect of compounds that may act as Mg^2+^ chelators and disrupt or inhibit the Obg_GC_ GTPase activity was tested using common chelators including EDTA (Amresco, Solon, OH), ethylene glycol tetra acetic acid (EGTA; Alfa Aesar, Ward Hill, MA), Nitrilotriacetic acid (NTA; TCI, Portland, OR), and citric acid (CA; Sigma Aldrich, St. Louis, MO) at the test-compound concentration used in HTS (40 μM). The protein was pre-incubated with each chelator for 1 h prior to the addition of GTP, and the assay was completed as described above.

### Primary screen

For the HTS, reagents were added to 384-well microplates using a BioTek MultiFlo Dispenser (BioTek, Shoreline, WA), and plates were delivered to the Oregon Translational Research and Drug Development Institute (OTRADI) satellite lab at Oregon State University for compound loading. Daughter plates were made from stock mother plates from the SIGA chemical library [7] in 10% DMSO, such that the final concentrations of the compounds and DMSO in the screening reaction were 40 μM and 0.8% (v/v), respectively. Each plate contained RM without Obg_GC_ (column 1), negative control comprising of RM and no compounds (column 2), and two columns of a positive control (RM without MgCl_2_, column 23–24).

Following compound addition (columns 3–22), 5 μM Obg was loaded into the wells, and the protein was allowed to incubate with compounds for 1 h at room temperature, prior to the addition of GTP (250 μM). The amount of released phosphate was measured following incubation as described above. The percent activity of each compound was calculated from the absorbance data using the following equation:

$$\%\;Activity=(\frac{C\text{-}Pc}{Nc\text{-}Pc})*100$$ Eq (1)

where *C* is the absorbance recorded for each reaction containing individual compounds and Obg_GC_, *Pc* is the average of all positive controls, and *Nc* is the average of all negative controls.

To confirm the inhibitory effect on GTPase activity of Obg, compounds resulting in 15% or more reduction in activity were selected for repeat screening with the BIOMOL Green assay in triplicate. Subsequently, compounds demonstrating 50% Obg_GC_ inhibition were selected for follow-up dose-dependence studies.

Three compounds selected for further testing included: Compound A: 2-chloro-4-nitro-6-{[(E)-2-nitroethenyl]amino}phenol (ChemBridge, San Diego, CA), Compound B: 9-(4-nitrophenyl)-5,13-disulfanyl-2-oxa-4,6,12,14-tetraazatricyclo[8.4.0.0^³,⁸^]tetradeca-1(10),3,5,7,11,13-hexaene-7,11-diol (Vitas-M Lab Ltd, Moscow, Russia), and bacterial GTPase EngA inhibitor, Garcinol (Enzo Life Sciences, Farmingdale, NY), identified in a previous HTS [43]. Obg_GC_ was incubated with 40 or 100 μM of the chemical for 1 h in RM prior to addition of GTP, followed by measurement of free phosphate amounts as described above.

### Secondary screen

N-methyl-3’-*O*-anthranoyl-(mant)-guanine nucleotide analogs of GTP and GDP (mant-GTP and mant-GDP, respectively) were purchased from Thermo Fisher Scientific (Waltham, MA). Protein (Obg_GC_ or *Obg_GC_, 2 μM) was incubated with either mant-GTP or mant-GDP nucleotide (0.3 μM) in buffer containing 50 mM Tris, 2 mM DTT, 1 mM EDTA, 50 mM KCl, 10% (wt/vol) glycerol, and for reactions with mant-GTP, 5 mM MgCl_2_ was included [11]. All fluorescence measurements were performed at 37°C using a Synergy HT plate reader (BioTek, Shoreline, WA). These studies were carried out at least eight times over multiple days. The means for the biological replicates with corresponding standard error of the mean (SEM) are reported.

### Assessment of the potential broad-spectrum activity of lead compounds

The activity of Obg_KP_ and Obg_MRSA_ was determined in the colorimetric and fluorescent assays as described for Obg_GC_. In addition, the GTP hydrolysis rate of both proteins (16 μM) was determined by recording the decrease in fluorescence of mant-GTP (0.3 μM) at 1 min intervals for 3 h [11]_._ Data were fitted to a single exponential decay equation, and the single turnover rate constant and the half-life of hydrolysis were calculated.

### Statistical analyses

Statistical analyses were conducted using GraphPad Prism 6.0 (GraphPad Software, La Jolla, CA). Unpaired t-test or One-way Analysis of Variance (ANOVA) with post hoc tests were used to determine differences in free phosphate detection or changes in fluorescence where appropriate (P ≤ 0.01). The primary screen was evaluated using statistical parameters of signal to noise (S:N), signal to background (S:B), signal window (SW), Z’ factor, and inter/intraplate variability (%CV), which were calculated as previously described [44, 45].

## Results and Discussion

Targeting ribosome function and biogenesis has led to some of the most clinically effective antibiotics currently in use, including tetracyclines and aminoglycosides [10, 33]. The association between Obg and the bacterial ribosome, specifically with the 50S subunit, has been well documented (reviewed in: [12]). In addition to its role in ribosomal maturation, alterations of many other critical cellular processes have also been attributed to the loss of Obg function, including DNA synthesis and replication, chromosomal segregation, regulation of the cell stress response, and bacterial persistence [11, 23–30]. The pivotal function(s) of Obg in key physiological processes make Obg a promising target for the development of novel antibiotics. Here we optimized conditions for HTS of small molecule inhibitors using as a model system Obg_GC_ from *N*. *gonorrhoeae*, a clinically important bacterium rapidly acquiring antibiotic resistance and causing multi-drug resistant infections that call for alternative antimicrobial interventions.

### Rationale and assay optimization

To develop an assay for screening of Obg inhibitors, we targeted the GTPase Obg activity because it is critical for Obg function *in vivo* and bacterial viability [46]. Measuring released phosphate as a result of Obg GTP hydrolysis also provides a technically feasible endpoint. The BIOMOL^®^ Green assay is a commercial version of a common phosphate detection assay originally established based on the principle that malachite green complexed with phosphomolybdate leads to a shift in λ_max_ [42]. A similar methodology was recently used in a HTS designed for a ribosome-associated bacterial GTPase, EngA, resulting in four prospective inhibitors [43]. One of the four, Garcinol, was available for purchase and tested for its ability to inhibit Obg_GC_ GTP hydrolysis. Up to 100 μM Garcinol, significantly higher than the IC50 reported for EngA (14.3 μM), was unable to inhibit the Obg_GC_ activity (S2A Fig), emphasizing the need to establish a new screening campaign to target the GTPase Obg.

An alternative means of measuring Obg activity is via fluorescent N-methyl-3’-*O*-anthranoyl guanine nucleotide analogs of GTP and GDP. Mant-GTP and mant-GDP have been widely utilized for examining the nucleotide binding and GTP hydrolysis of various G-proteins, including Obg homologs from *C*. *crescentus* [31], *E*. *coli* [47], *V*. *harveyi* [48], and recently *N*. *gonorrhoeae* [11]. When bound to proteins, fluorescence increases by ~2- and 1.3-fold for mant-GTP and mant-GDP, respectively. However, despite the sensitivity of the fluorescent assay, the minimal fold induction does not provide a substantial window for HTS [44, 45]. Therefore, we proceeded with optimizing the BIOMOL Green assay for efficiency and high-throughput competency.

**Fig 1:**
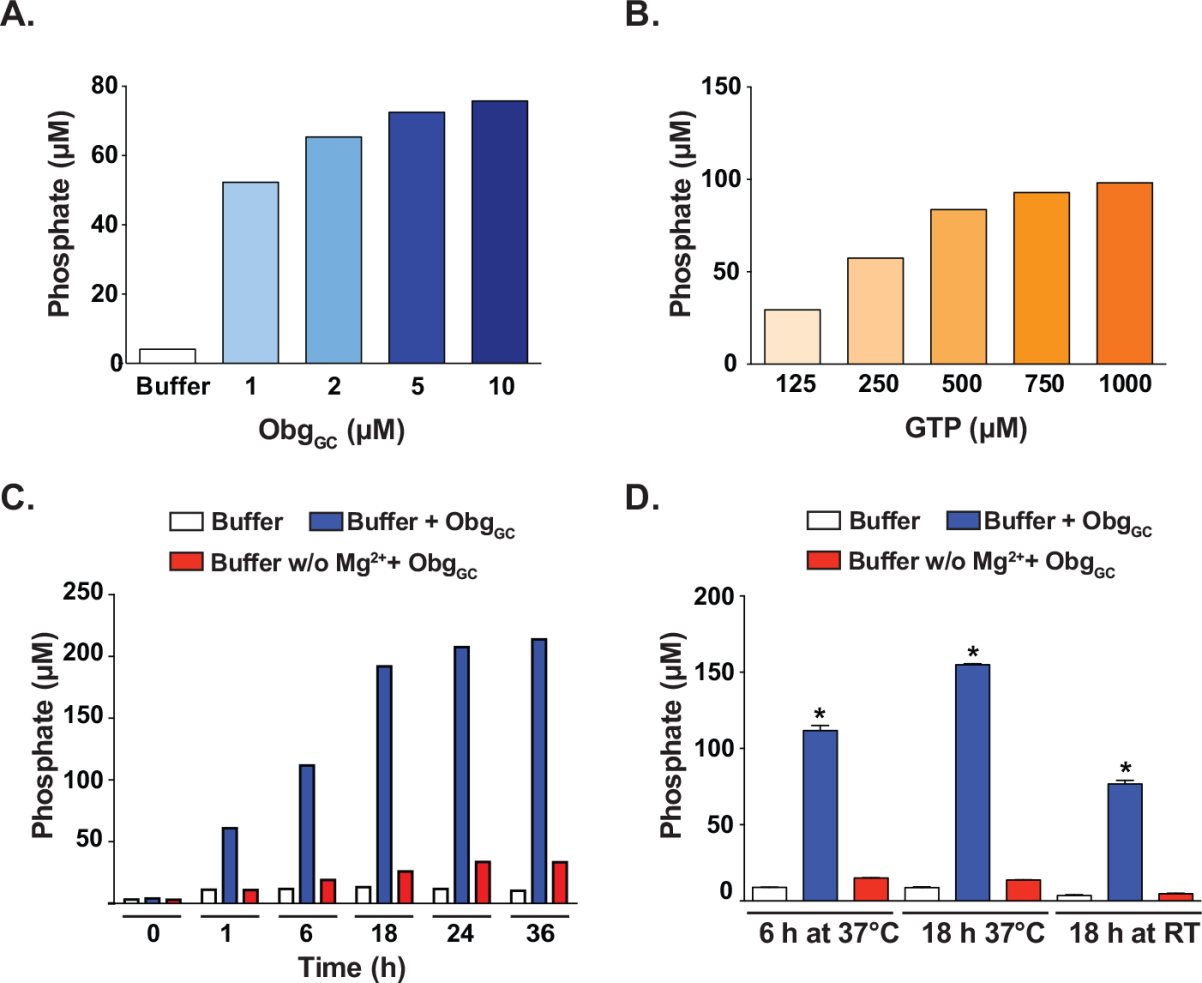
Assay optimization. The concentrations of Obg_GC_ and GTP were selected to achieve an optimal signal window for the HTS assay using as a read-out colorimetric free phosphate quantitation with BIOMOLGreen. **(A)** Various concentrations of Obg_GC_ (1–10 μM) were incubated with 125 μM GTP for 6 h at 37°C, followed by measurement of free phosphate amounts. **(B)** Obg_GC_ (5 μM) was incubated with increasing concentrations of GTP (125–1000 μM) for 6 h at 37°C and the free phosphate present in each reaction was quantitated. **(C)** Time course with 5 μM Obg_GC_ and 250 μM GTP assayed at 0, 1, 6, 18, 24 and 36 h demonstrated that the Obg-dependent GTP hydrolysis continued to increase until approximately 18 h. **(D)** Obg_GC_ (5 μM) was incubated with GTP (250 μM) in the presence or absence of Mg^2+^ for 6 or 18 h at either 37°C or room temperature (RT). The signal windows for the three incubation conditions were 2.33, 4.25, and 2.76, respectively, indicating that 18 h incubation time at 37°C was the most optimal. There was no significant difference between free phosphate amounts detected in reactions lacking Mg^2+^ (red bars) and that of buffer alone (background, white bars). Asterisk denotes significant difference between complete reaction (blue bars) and reaction mixture lacking Mg^2+^ (ANOVA, P < 0.0001).

The initial conditions used to optimize the assay, the protein and GTP concentrations as well as incubation time, were chosen with the knowledge that Obg_GC_ hydrolyzes GTP approximately twenty times slower than the *V*. *harveyi* Obg and two-fold slower than that of *C*. *crescentus* Obg, and slightly slower than *E*. *coli* Obg protein, with a half-life of GTP of approximately 50 min [11]. Preliminary studies using the BIOMOL Green assay revealed that at least 6 h of incubation were required to provide a significant fold change above background (data not shown). We therefore began optimization studies with a 6 h incubation length to accommodate for the slow GTP hydrolysis time. In contrast, incubation time for the EngA GTPase HTS was only 25 min [43]. When Obg_GC_ was incubated with 125 μM GTP for 6 h at 37°C, the amount of phosphate detected in the presence of 5 and 10 μM Obg_GC_ showed a marginal difference, 72 versus 75 μM, and therefore 5 μM was selected to obtain a more favorable protein to compound ratio in the screen (Fig 1A). Incubation of 5 μM Obg_GC_ with 125–1000 μM GTP, showed a linear dependence on substrate concentration up to 250 μM, and it was therefore selected as the GTP concentration for the screen (Fig 1B).

While the 6 h incubation provided a reliable signal window enabling detection of potential inhibitors, the feasibility of the HTS necessitated an overnight incubation. For instance preparation of thirty 384-well plates, our desired goal per day, required approximately 6–7 h of handling prior to the addition of GTP and assay incubation. In addition, BIOMOL Green reagent required a 30 min incubation and each assay plate took approximately 3 min to read on the spectrophotometer (S1 Table). Further, we wanted to avoid preparation of assay plates ahead of time and subsequent freeze thawing of compound libraries. To mitigate the impractical timeline, we performed a time course, measuring phosphate release following 1, 6, 18, 24, and 36 h of incubation (Fig 1C). We observed that the reaction continued to increase between 6 and 18 hrs. To further compare the two time points, the amounts of released phosphate were tested under multiple incubation conditions: for 6 h at 37°C, 18 h at 37°C, and 18 h at room temperature for comparison, in triplicate 384-well plates which would allow us to obtain preliminary HTS statistical analyses (Fig 1D). Ultimately, 18 h at 37°C was selected because the greatest amplitude of signals between the positive and negative controls was achieved, as demonstrated by the signal window of 4.25, compared to 2.33 and 2.76 for 6 h at 37°C and 18 h at room temperature, respectively.

Our positive control for the assay was reaction mixture lacking Mg^2+^, because inhibitors of Obg are currently not known. The negative control was the complete reaction in buffer A (as described in Materials and Methods) containing Obg_GC_, GTP and 10 mM MgCl_2_. For all tested incubation times, there was a statistically significant difference between the amounts of free phosphate detected in reactions where Mg^2+^ was present as compared to reactions without Mg^2+^ (Fig 1C and 1D; blue bars versus red bars).

The dependence of Obg proteins on Mg^2+^ to bind GTP has been previously demonstrated, and in GC, the maximal formation of Obg-GTP complexes occurs between 5 and 10 mM Mg^2+^ [11, 31, 47, 48]. In contrast, binding of GDP by Obg does not require Mg^2+^, and in fact was inhibited for Obg_GC_ at concentrations above 1 mM [11], suggesting that our assay reaction conditions were conducive to multiple turnover, and our positive control represented an optimally inhibited reaction. In contrast, for GTPases like Ras GAPs, G_αi1_, and GDP/GTP exchange are rate limiting and the enzyme turnover rate is slow. Therefore utilizing a steady-state assay like BIOMOL Green with such proteins becomes a challenge [12, 49, 50].

The kinetic characterization of Obg_GC_ demonstrated a Michaelis-Menten constant (Km) of 78 μM (S3 Fig). Using a concentration of substrate at or less than the Km of the enzyme has been suggested to create conditions sensitive to competitive inhibitors in HTS [43, 45, 51]. However, given the proposed Obg’s intracellular action as a GTP sensor [12], and it’s GTP/GDP binding affinity under different Mg^+2^ concentrations [11], using a higher concentrations of GTP provided a wider signal window to identify potential inhibitors. The capacity of a HTS to detect an inhibitor depends not only on the biochemical mechanism of action between the enzyme and the substrate, but also the concentration of the inhibitor and the mechanism of action of an inhibitor [52]. Therefore, the concentration of library compounds used in our screen was 40 μM to increase the chance of detecting an inhibitor, regardless of the mechanism(s) of action.

### Assessment of Obg_GC_ solvent tolerance and chelator challenge

**Fig 2:**
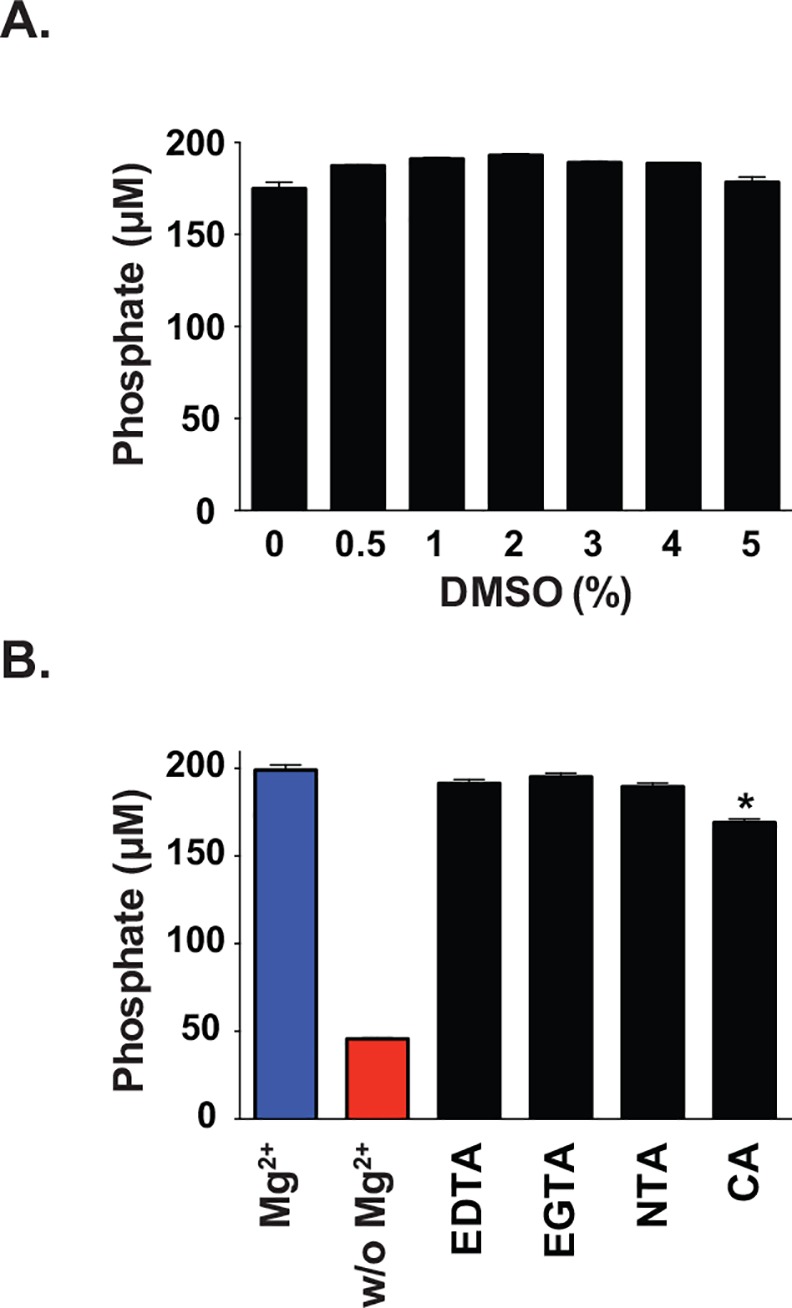
Assessments of solvent tolerance and chelator challenge. Potential complications of screening conditions were addressed by challenging Obg_GC_ with various concentrations of DMSO **(A)** or multiple known chelators **(B)** followed by measurement of GTPase Obg_GC_ activity using free phosphate quantitation with BIOMOL Green. Protein activity was similar in the presence of 0–5% DMSO and in the presence of three common chelators EDTA, EGTA, and NTA tested 40 μM final concentration. CA significantly decreased the amount of phosphate detected, but it also decreased the background phosphate in the reaction lacking Obg_GC_ (not shown), and was therefore altering the absorbance of the reagent, not acting as a chelator. Asterisk denotes significant difference (ANOVA, P < 0.0001).

The inherent range of solubility of chemicals in any screening library necessitates the use of a solvent in biochemical assays. To assess the Obg_GC_ tolerance to the compound solvent, the Obg-GTPase activity was examined using 0, 1, 2, 3, 4, and 5% DMSO during optimized conditions for 18 h at 37°C as described above (Fig 1C). The amount of released phosphate remained similar (~180 μM) under all tested conditions, providing evidence that the GTPase activity of Obg_GC_ was not affected by the presence of solvent to be used in our screen (Fig 2A).

Chelators are often used in biochemistry to sequester metals in reactions, rendering the metals biologically unavailable. Common chelators including EDTA, EGTA, and NTA exhibit varying capacities to bind transition metals such as Ca^2+^, Mg^2+^, Fe^2+^, and Fe^3+^. The potential for other molecules to exhibit some ability to bind biologically relevant metals, including Mg^2+^, a critical component of our assay because of its requirement for Obg-GTP interaction, may pose a challenge to our study design, despite that the concentration of Mg^2+^ in the buffer (10 mM) is in excess of the 40 μM compounds. Therefore, the ability for common chelators to inhibit Obg_GC_ activity under the experimental conditions of the primary screen was assessed. Obg_GC_ was pre-incubated for 1 h with 40 μM of different chelators including EDTA, EGTA, NTA and CA. Only the presence of CA caused a significant decrease in the amount of free phosphate detected, equivalent to less than 20% inhibition (Fig 2B). However, the background phosphate levels for CA (chelator + buffer A + GTP) was 50% lower than that of the other three chelators (data not shown), suggesting that the reduction in signal was due to an interaction with a component of the reagents used, not a *bona fide* inhibition of protein activity.

Together, these studies demonstrated the compatibility of our assay with a high DMSO concentration as well as with a potential chelating agent.

### Primary screen for inhibitors of Obg_GC_

Our assay development efforts afforded primary screening conditions that were both sensitive and practical for HTS. Therefore, Obg_GC_ was screened against 40,000 diverse compounds using the SIGA chemical libraries through OTRADI. These libraries contain an assortment of both synthetic molecules and natural products, and have been previously used in other HTS [7, 53, 54].

An initial screen of 3,200 compounds (10 plates) was performed, and the calculated Z’ value was 0.55 and a signal window of 4.47, similar to that of our optimization studies (Fig 1C), indicating an excellent assay design [44, 45]. The entire pilot screen took place over five weeks, and the performance of our assay was assessed with standard HTS statistical parameters [44, 45] on each day of the screen (Table 1). The averaged Z’ factor for the entire screen was 0.58 ± 0.02. The intra- and inter-plate (%CV) variability was also determined for both positive and negative controls. Inter-plate variability was higher for both negative (9.32 ± 0.6%) and positive (13.13 ± 2.9%) controls than the respective intra-plate variabilities (3.58 ± 1.4% and 5.13 ± 1.6%). Variability among the positive controls was on average higher than that of negative controls for both statistical parameters (%C), which may be at least partially due to the fact that there were twice as many positive controls as there were negative controls. Overall, the statistical evaluation of our screen showed a robust assay suitable for HTS of small molecules libraries for identification of potential Obg inhibitors.

**Table 1:** Statistical analysis of assay performance.

# compounds screened	S:N^a^	S:B^b^	SW^c^	Z’^d^	% nCV_inter_^e^	% nCV_intra_^f^	% pCV_inter_^g^	% pCV_intra_^h^
3,200	34.67	4.98	4.47	0.55	9.78	5.46 ± 2.3	11.49	6.30 ± 1.2
12,800	27.94	5.81	5.55	0.58	8.65	2.51 ± 1.1	17.21	4.51 ± 4.6
12,800	44.08	5.7	4.67	0.57	10.02	2.06 ± 0.8	10.67	2.70 ± 1.5
3,520	29.59	5.51	5.48	0.58	8.67	3.28 ± 2.0	15.24	5.49 ± 2.6
7,680	46.51	6.13	5.27	0.6	9.47	4.61 ± 0.7	11.04	6.64 ± 3.1
*Average* ± *SD*	36.56 ± 8.4	5.63 ± 0.4	5.09 ± 0.5	0.58 ± 0.02	9.32 ± 0.6	3.58 ± 1.4	13.13 ± 2.9	5.13 ± 1.6

^a^Signal to noise ratio is a measure of signal strength

^b^Signal to background ratio

^c^Signal Window is a measure in the difference in signal between negative and positive controls

^d^Z-factor is a measurement of the performance of the assay under the defined conditions

^e,f^ Inter- and Intra-plate, respectively, variability for negative control.

^g,h^ Inter- and Intra-plate variability, respectively, for positive control.

Our pilot screening campaign revealed 69 compounds exhibiting greater than 15% inhibition of Obg, or less than 85% residual activity, as calculated using Eq 1 explained in the Materials and Methods section. Screening 40,000 compound multiple times to improve assay sensitivity [52] was cost prohibitive. Therefore, for cherry-picking confirmation, molecules with 15% and greater inhibition in the primary screen were retested at 40 μM in triplicate. Ultimately, a hit was defined as a chemical resulting in 50% or less of residual Obg activity compared to the negative control calculated using Eq 1. Two chemicals, named Compound A and Compound B (defined in Materials and Methods), which inhibited Obg_GC_ activity approximately 50% on three replicate plates, were selected for further analysis. Both compounds were ordered from outside vendors, and their ability to inhibit Obg_GC_ was tested at 40 and 100 μM, alongside the EngA inhibitor, Garcinol, identified in [43]. To eliminate the possibility that these compounds were false positives, the signal produced by 40 μM phosphate in the presence of 40 and 100 μM compound was also measured. Neither of the lead compounds demonstrated any significant inhibition of the protein, despite having a 50% inhibition in three replicate plates of the follow-up screen (S2B and S2C Fig).

The low hit rate following our primary screen, and lack of confirmed lead compound, is not uncommon for a pilot screen of only 40,000. In other HTS studies, > 85% of the initial hits were found to be non-specific for the target molecule [43, 53, 54]. Screening a greater number of small molecules will increase the chance of identifying candidates for further development. Ultimately, two inhibitors were identified through our screen, which demonstrates that the assay is working. The failure of confirmation testing of the initial hits using purchased chemicals is not a reflection on the assay, but may suggest that their anti-Obg activity originated from a degradation product(s) and/or impurities of the parent compound sample in the library. There is a wide range of rearrangements and chemical reactions (e.g. oxidation, hydrolysis, isomerization) that may occur in a structurally diversified collection of compounds upon storage in DMSO solution [55]. Other factors such as compound concentration, intrinsic stability, presence of reactive contaminants, and storage related factors (e.g. type of containers, storage conditions, time of storage) could affect compound stability [56].

### Secondary screen for elucidating mechanism of Obg_GC_ inhibition

A secondary assay was developed as a follow up study for lead compounds in order to not only confirm their ability to inhibit Obg hydrolysis, but also to elucidate a possible mechanism of compound action. When fluorescent guanine nucleotide analogs, mant-GTP and mant-GDP, are bound to Obg_GC_, there is 1.9- and 1.3-fold increase in fluorescence, respectively [11], which is similar to values reported for other bacterial Obg proteins [31, 47, 48]. Overall, this assay is useful because it delivers a different read-out method with the sensitivity inherent to a fluorescence assay (compared with the colorimetric reaction used in the primary screening) and provides a means to dissect the interaction(s) that may occur between a compound, Obg, and mant-nucleotides.

**Fig 3:**
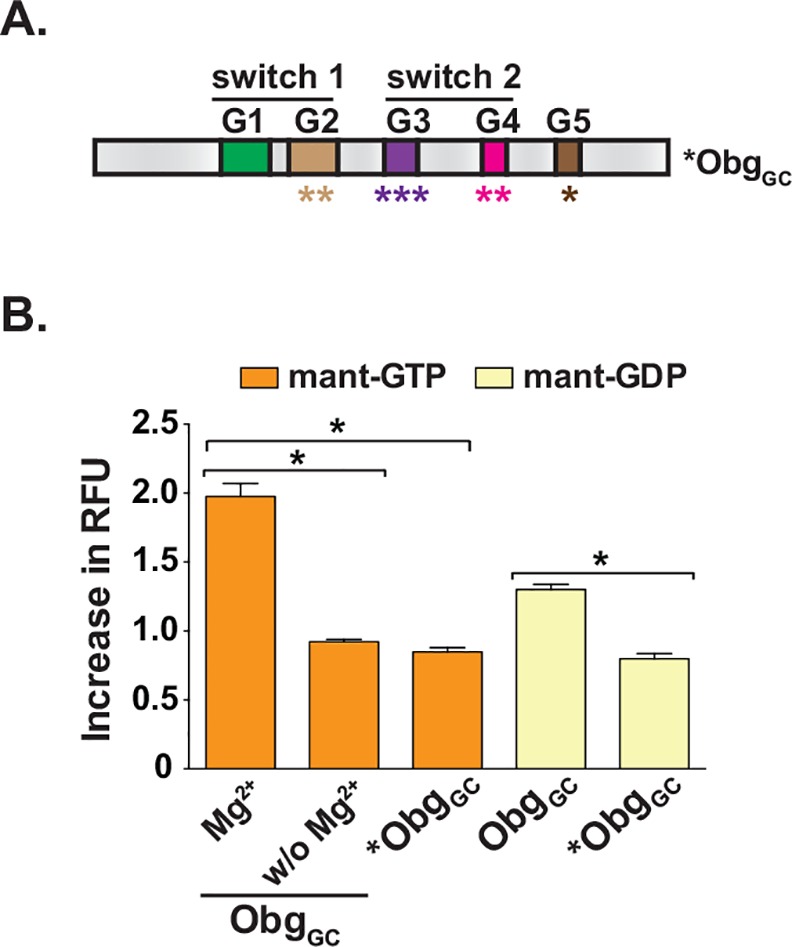
Secondary assay design. **(A)** The domain architecture of Obg with switch 1 and switch 2 are shown. Five conserved G motifs (G1-G5) are indicated in colored boxes. The mutated variant of Obg_GC_, *Obg_GC_, unable to interact with Mg^2+^, GTP, and GDP, was designed using as a template the *obg* gene from *N*. *gonorrhoeae* FA1090. The altered amino acids residues include T192S, T193S, A212L, D213E, P215H, N284Q, K285R, S329V within G domains (outlined in detail in S1 Fig). **(B)** Assessment of mant-nucleotides binding by wild type (Obg_GC_) and mutated (*Obg_GC_). An increase in Relative Fluorescence Units (RFU) of mant-GTP (orange bars) and mant-GDP (yellow bars) upon addition of different Obg_GC_ variants was measured in at least eight experiments performed on separate occasions and averages with corresponding SEM are presented. *Obg_GC_ activity in the mant-GTP assay was not significantly different from that of Obg_GC_ in the absence of Mg^2+^. Asterisk denotes significant difference (ANOVA, P < 0.0001).

In addition to the positive control of reaction mixture lacking Mg^2+^, used in the primary screen assay, an alternative positive control was developed for the secondary assay. An Obg variant, *Obg_GC_, was designed with multiple mutations within the G domain, at T192S, T193S, A212L, D213E, P215H, N284Q, K285R, and S329V (Fig 3A and S1 Fig), rendering a protein that was no longer capable of binding GTP or GDP (Fig 3B). An additional Obg_GC_ mutant, Obg_GC_-T192AT193A, has a double alanine substitution for the two conserved tyrosine residues in the G2 motifs, 192 and 193, that are required for binding Mg^2+^ [11]. These mutations generate a protein that is unable to bind GTP, while retaining its ability to bind GDP. Utilizing this mutant in the secondary assay could provide additional insight into the potential mechanism of Obg_GC_ inhibition by a lead compound.

### Assessment of broad-spectrum potential of Obg inhibitors

The ability of potential lead compounds to act on other clinically relevant bacteria by targeting Obg could be further assessed by utilizing Obg homologs from bacterial species associated with serious antibiotic resistant infections for instance *K*. *pneumoniae* and MRSA [57–59]. *K*. *pneumoniae* is a Gram-negative bacterium and an opportunistic pathogen, which causes, in addition to pneumonia, infections in the urinary tract, wound or surgical sites, blood stream, and meningitis. It is one of the top organisms causing infections in hospitalized patients [57, 59]. Particularly vulnerable to *K*. *pneumoniae* infections are immunocompromised individuals, in which the disease can result in death. *K*. *pneumoniae* is one of the two most common carbapenem-resistant *Enterobacteriaceae*, and is listed by the CDC as an urgent threat as a result of its prevalence and resistance to antibiotics [2]. MRSA is a Gram-positive bacterium, which causes a range of illnesses, from skin and wound infections to sepsis, and it constitutes approximately 28% of hospital-acquired bacterial pneumonia [58, 59]. The number of MRSA infections is among the highest of all antibiotic-resistant diagnoses, and it is therefore listed as a serious threat by the CDC [2]. Presently, there are effective antibiotics capable of treating MRSA-derived infections. However, the prevalence and persistence of such infections may facilitate the resilience of more resistant strains of MRSA, generating a greater need for novel treatments [2]. Together with *N*. *gonorrhoeae*, they represent a diverse set of clinically important bacteria that are all in need of novel and aggressive antibiotic development.

**Fig 4:**
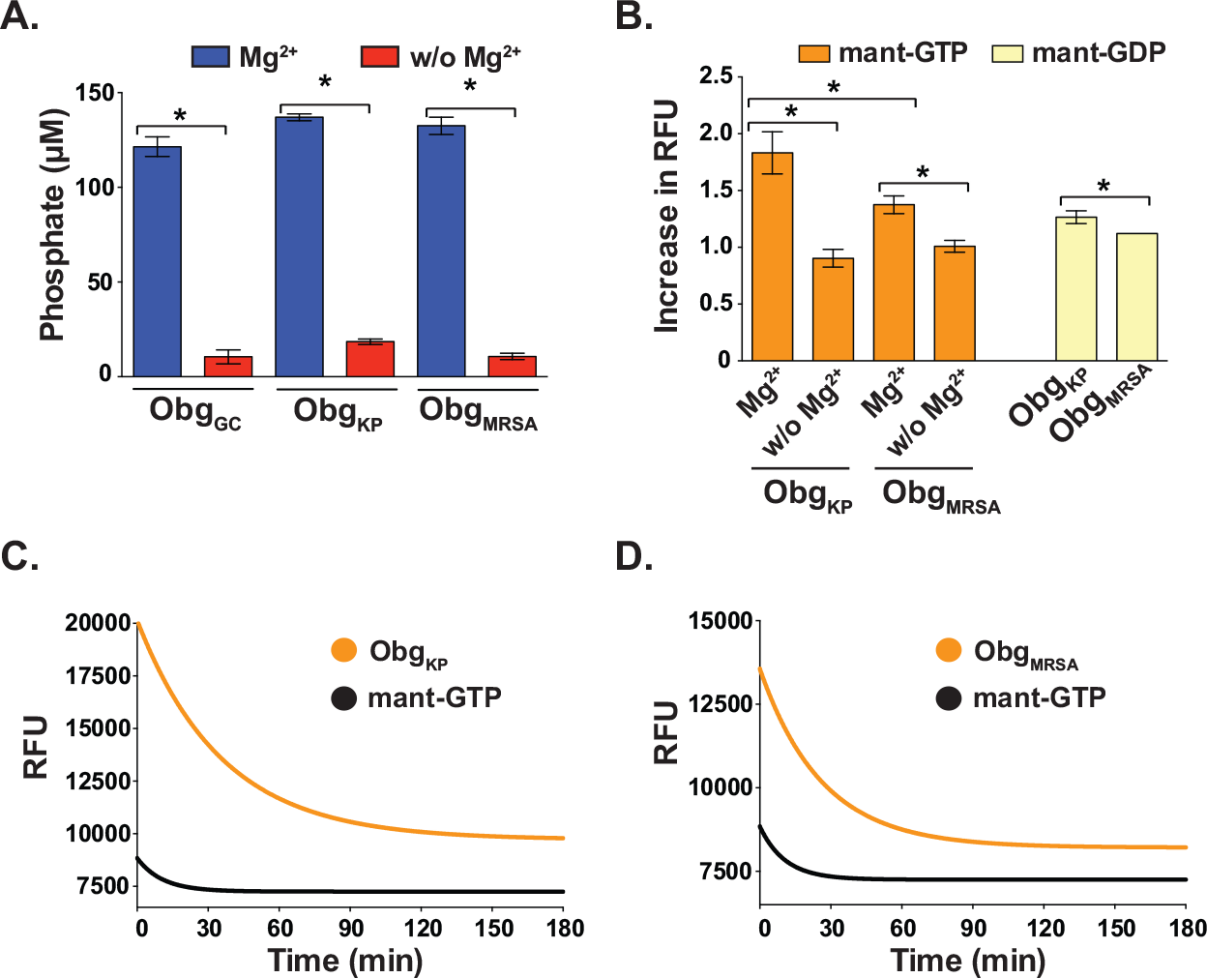
Biochemical analysis of Obg isolated from *K*. *pneumoniae* and MRSA. To evaluate the potential of broad-spectrum activity of lead compounds identified using Obg_GC_ as a target, Obg_KP_ and Obg_MRSA_ were purified and GTP hydrolysis as well as GTP and GDP binding were examined using the colorimetric (A) and fluorescence-based activity assays using mant-nucleotides (B, C and D). **(A)** The free phosphate detection assay was conducted with 5 μM protein and 250 μM GTP and the reactions were incubated for 18 h at 37°C followed by the addition of BIOMOL Green reagents and absorbance measurements. Similar concentrations of phosphate were detected with both Obg_KP_ and Obg_MRSA_ (not significantly different, t-test, P = 0.3109). Both proteins required the presence of Mg^2+^ to hydrolyze GTP (significantly different, respectively, T-TEST P< 0.0001). The data shows averages, with corresponding SEM, of six biological replicates. **(B)** Binding of mant-GTP (orange bars) and mant-GDP (yellow bars) to Obg_KP_ and Obg_MRSA_ was examined in the presence and absence of Mg^2+^, as indicated below the graph. Obg_KP_ binding of mant-GTP and mant-GDP increased the RFU ~1.8- and 1.3-fold, respectively. Whereas ~1.4- and 1.1-fold increase in RFU was observed for the corresponding mant-nucleotides upon binding by Obg_MRSA_. There was a significant difference in ability of Obg_KP_ and Obg_MRSA_ to bind mant-GTP (t-test, P <0.001). The reliance on Mg^2+^ for both proteins was demonstrated by the absence of an increase in RFU in reaction buffer deficient in Mg^2+^ (Significantly different, respectively, t-test, P< 0.0001). The data shows averages with corresponding SEM of five experiments performed on separate occasions. Hydrolysis of mant-GTP by Obg_KP_ **(C)** and Obg_MRSA_ **(D)** was monitored by recording the decrease in fluorescence that is coupled to the conversion of mant-GTP-Obg to mant-GDP-Obg complexes over 3 h. The fluorescence intensity of the mant-GTP in the absence of protein served as a control and is shown in black. The first-order rate constant, k_*h*_, of 4.64 × 10^−4^s^−1^ and 6.36 × 10^−4^s^−1^, and half-life (T_1/2_) of 24.88 min and 18.17 min, were calculated for Obg_KP_ and Obg_MRSA_, respectively.

The Obg proteins of *K*. *pneumoniae*, Obg_KP_, and MRSA, Obg_MRSA_, were cloned, expressed in *E*. *coli* and purified, followed by characterization of their biochemical activities in colorimetric and fluorescent assays under the same conditions described for Obg_GC_ (Fig 4). All three proteins exhibited similar GTPase activity in the BIOMOL Green assay, producing ~120, 140, and 130 μM phosphate for Obg_GC_, Obg_KP_, and Obg_MRSA_, respectively, with 5 μM protein and 250 μM GTP in each reaction at the end of the 18 h incubation (Fig 4A). Like Obg_GC_, Obg_KP_ and Obg_MRSA_, hydrolyzed GTP only in the presence of Mg^2+^. Obg_KP_ also behaved similar to other Obg homologs [11, 31, 47, 48] in the fluorescence-based assay, as binding of mant-GTP and mant-GDP resulted in ~1.8- and 1.3-fold increase in fluorescence, respectively (Fig 4B). In contrast, complexes of Obg_MRSA_-mant-GTP and Obg_MRSA_-mant-GDP showed ~1.4- and 1.1-fold elevation in relative fluorescence, respectively (Fig 4B). There was, however, no significant difference in the amount of free phosphate detected using the BIOMOL Green assay at the end of 18 h incubation with Obg_KP_ and Obg_MRSA_ (ANOVA, P = 0.3109), suggesting that the Obg_MRSA_ may not bind mant-nucleotides as readily as other Obg homologs.

Another useful parameter to further characterize the mechanism of action of a lead compound molecule is the assessment of the GTP hydrolysis rate of Obg proteins. Therefore, we next examined the GTPase activity of the Obg_KP_ and Obg_MRSA_ by monitoring the decrease in fluorescence that is associated with the single-turnover conversion of bound mant-GTP to bound mant-GDP. The reduction in fluorescence was fitted with a single exponential decay equation, and first-order rate constants, k_*h*_, 4.64 × 10^−4^s^−1^ and 6.36 × 10^−4^s^−1^, as well as half-lives (T_1/2_) of 24.88 min and 18.17 min, were determined for Obg_KP_ and Obg_MRSA_, respectively. The hydrolysis rate of both proteins were similar to Obg from *C*. *crescentus*, approximately 2-fold faster than *N*. *gonorrhoeae* and *E*. *coli*, and 11 and 8-fold slower than *V*. *harveyi*, respectively [11, 31, 47, 48]. The slight variations in the binding and hydrolysis activities of the Obg homologues may reflect species-specific subtle differences in Obg functions. Nevertheless, combining both the colorimetric and fluorescent assays provide powerful tools for elucidating Obg inhibitors.

## Conclusions

Obg protein appears to be an excellent target for the development of novel antimicrobials because of its essential nature to both Gram-positive and Gram-negative bacteria. Accordingly, we have optimized an inexpensive, statistically sound, high-throughput assay to screen for inhibitors of Obg_GC_. Further, we have developed a secondary method to evaluate the ability of potential lead compounds to interfere with Obg-nucleotide binding and dissect a potential mechanism of action. Finally, we propose to employ Obg homologs from other clinically important and very different bacterial species, *K*. *pneumoniae* and MRSA, to test the potential for broad-spectrum antibiotic development in targeting Obg.

The screening approach described here will be useful in identifying inhibitors of a ribosome-associated protein with a proposed role in bacterial multidrug tolerance that will further the advancement of broad-spectrum therapeutics, thus meeting the needs raised by CDC and WHO.

## Supporting Information

S1 Fig Schematic outline of Obg_GC_ architecture with introduced mutations. The individual domains of Obg_GC_ are labeled in dark blue. The central, GTP-binding domain includes switch I and switch II and five conserved G motifs (G1-G5; indicated in blue boxes). The introduced substitutions within the G motifs are designated in red.
(DOCX)

S2 Fig Evaluation of inhibitory potential of GTPase EngA inhibitor, Garcinol, and potential lead compounds identified in a pilot Obg_GC_ screen, A & B, using the Biomol® Green assay. Obg_GC_ (5 μM) was pre-incubated with tested compounds (40 and 100 μM) followed by the addition of GTP (250 μM), incubation 18 h at 37°C and free phosphate measurement.
(DOCX)

S3 Fig Kinetic characterization of Obg_GC._ The Km of Obg_GC_ was determined using 5 μM Obg_GC_ and a range of GTP from 10–1000 μM over 6 h. The Km for Obg_GC_ was determined to be 78.76 μM.
(DOCX)

S1 Table Timeline of the Obg_GC_ HTS procedures. (DOCX)
