# Supplementary material for: Targeting an Essential GTPase Obg for the Development of Broad-Spectrum Antibiotics
Source: PLoS One. 2016 Feb 5;11(2):e0148222. doi: 10.1371/journal.pone.0148222 (PMC4743925; doi:10.1371/journal.pone.0148222)
Supplement: S1 Fig — The individual domains of ObgGC are labeled in dark blue. The central, GTP-binding domain includes switch I and switch II and five conserved G motifs (G1-G5; indicated in blue boxes). The introduced substitutions within the G motifs are designated in red. (DOCX) [file pone.0148222.s001.docx]

**Supplemental Information S1 Fig**.
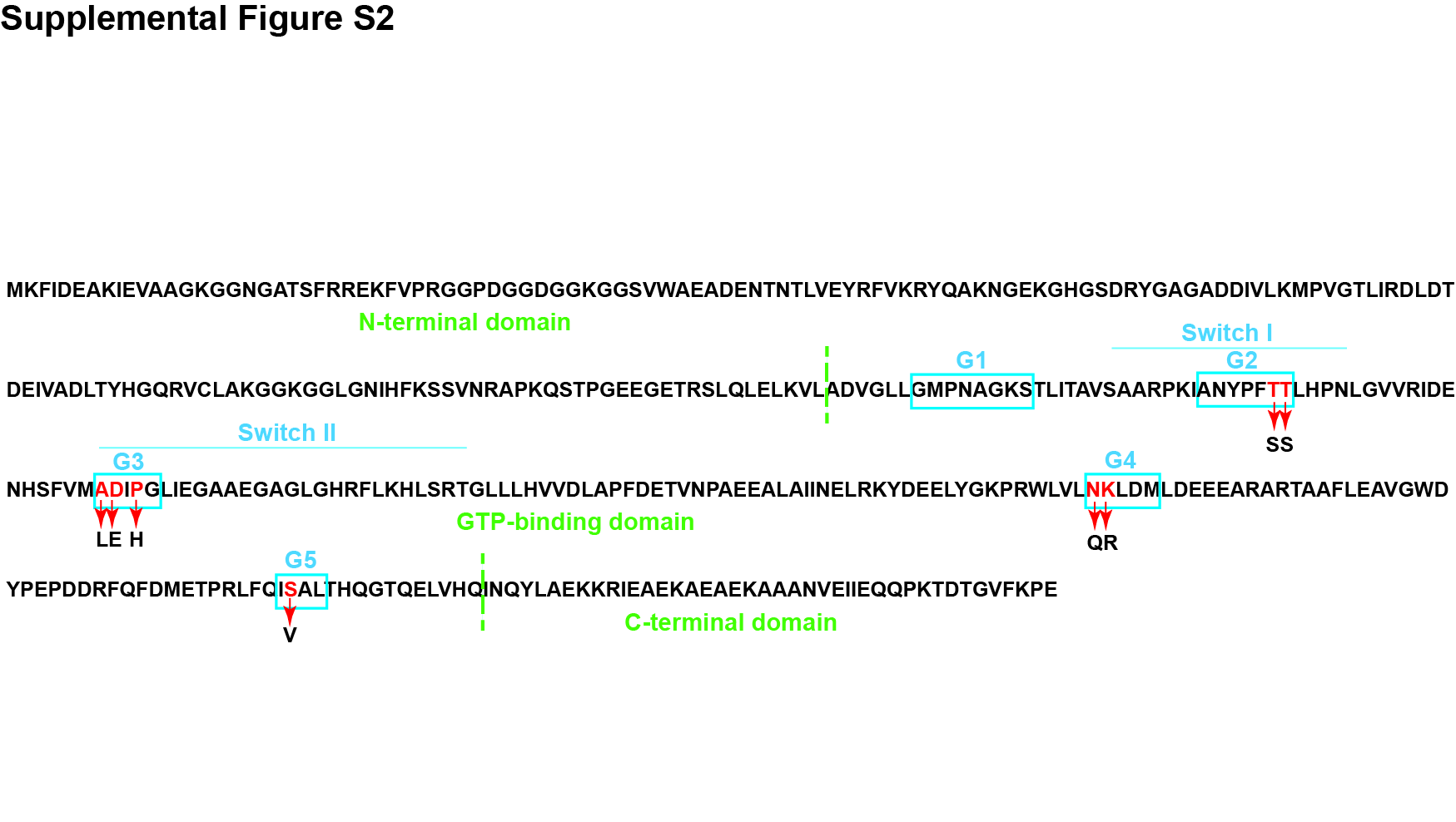


**S1 Fig**. Schematic outline of ObgGC architecture with introduced mutations. The individual domains of Obg_GC_ are labeled in dark blue. The central, GTP-binding domain includes switch I and switch II and five conserved G motifs (G1-G5; indicated in blue boxes). The introduced substitutions within the G motifs are designated in red.
