# Supplementary material for: Targeting an Essential GTPase Obg for the Development of Broad-Spectrum Antibiotics
Source: PLoS One. 2016 Feb 5;11(2):e0148222. doi: 10.1371/journal.pone.0148222 (PMC4743925; doi:10.1371/journal.pone.0148222)
Supplement: S2 Fig — ObgGC (5 μM) was pre-incubated with tested compounds (40 and 100 μM) followed by the addition of GTP (250 μM), incubation 18 h at 37°C and free phosphate measurement. (DOCX) [file pone.0148222.s002.docx]

**Supplemental Information S2 Fig**.

A.

B.

C.

**S2 Fig.** Evaluation of inhibitory potential of GTPase EngA inhibitor, Garcinol **(A)**, and potential lead compounds identified in a pilot Obg_GC_ screen, A and B, **(B,C)** using the Biomol® Green assay. Obg_GC_ (5 µM) was pre-incubated with tested compounds (40 and 100 µM) followed by the addition of GTP (250 µM), incubation 18 h at 37 ºC and free phosphate measurement.
