# Supplementary material for: Targeting an Essential GTPase Obg for the Development of Broad-Spectrum Antibiotics
Source: PLoS One. 2016 Feb 5;11(2):e0148222. doi: 10.1371/journal.pone.0148222 (PMC4743925; doi:10.1371/journal.pone.0148222)
Supplement: S3 Fig — The Km of ObgGC was determined using 5 μM ObgGC and a range of GTP from 10–1000 μM over 6 h. The Km for ObgGC was determined to be 78.76 μM. (DOCX) [file pone.0148222.s003.docx]

**Supplemental Information S3 Fig**.

**S3 Fig.** Kinetic characterization of Obg_GC._ The Km of Obg_GC_ was determined using 5 μM Obg_GC_ and a range of GTP from 10-1000 μM over 6 h. The Km for Obg_GC_ was determined to be 78.76 µM.
