# Supplementary material for: Targeting an Essential GTPase Obg for the Development of Broad-Spectrum Antibiotics
Source: PLoS One. 2016 Feb 5;11(2):e0148222. doi: 10.1371/journal.pone.0148222 (PMC4743925; doi:10.1371/journal.pone.0148222)
Supplement: S1 Table — (DOCX) [file pone.0148222.s004.docx]

|  | **Protocol** |  |  | |  | | **Time (hrs)** | | |  |  | |  |
| --- | --- | --- | --- | --- | --- | --- | --- | --- | --- | --- | --- | --- | --- |
| 1 | Prepare & Load buffer into 30 plates | | | |  | | 1.5 | | |  |  | |  |
| 2 | Load chemicals/spin plates (~10 plates/h) | | | | | | 3.5 | | |  |  | |  |
| 3 | Load protein/spin plates | | |  | |  | | 0.5 |  | | |  | |
| 4 | Incubate protein with compounds | | | |  | | 1.0 | | |  |  | |  |
| 5 | Load GTP/spin plates | | | |  | | 0.5 | | |  |  | |  |
| 6 | Incubate at 37ºC | | | | | | 18 | | |  |  | |  |
| 7 | Biomol green (~10 plates at a time/incubation 30 min) | | | | | | 1.5 | | |  |  | |  |
| 8 | Read plates (2:48 min/read + loading = ~ 3 min/plate) | | | | | | 1.5 | | |  |  | |  |

**S1 Table. Timeline of the Obg_GC_ HTS procedures.**
